# The TARC/sICAM5 Ratio in Patient Plasma is a Candidate Biomarker for Drug Resistant Epilepsy

**DOI:** 10.3389/fneur.2012.00181

**Published:** 2013-01-03

**Authors:** John R. Pollard, Ofer Eidelman, Gregory P. Mueller, Clifton L. Dalgard, Peter B. Crino, Christopher T. Anderson, Elizabeth J. Brand, Evren Burakgazi, Sai K. Ivaturi, Harvey B. Pollard

**Affiliations:** ^1^Penn Epilepsy Center, Department of Neurology, University of PennsylvaniaPhiladelphia, PA, USA; ^2^Department of Anatomy, Physiology and Genetics, The Center for Medical Proteomics and Genomics, Uniformed Services University of the Health SciencesBethesda, MD, USA; ^3^Department of Neurology, Cooper Medical Center, University of Medicine and DentistryCamden, NJ, USA

**Keywords:** epilepsy, neuroinflammation, biomarkers

## Abstract

Epilepsy is a common affliction that involves inflammatory processes. There are currently no definitive chemical diagnostic biomarkers in the blood, so diagnosis is based on a sometimes expensive synthesis of clinical observation, radiology, neuro-psychological testing, and interictal and ictal EEG studies. Soluble ICAM5 (sICAM5), also known as telencephalin, is an anti-inflammatory protein of strictly central nervous system tissue origin that is also found in blood. Here we have tested the hypothesis that plasma concentrations of select inflammatory cytokines, including sICAM5, might serve as biomarkers for epilepsy diagnosis. To test this hypothesis, we developed a highly sensitive and accurate electrochemiluminescent ELISA assay to measure sICAM5 levels, and measured levels of sICAM5 and 18 other inflammatory mediators in epilepsy patient plasma and controls. Patient samples were drawn from in-patients undergoing video-EEG monitoring, without regard to timing of seizures. Differences were defined by *t*-test, and Receiver Operating Condition (ROC) curves determined the ability of these tests to distinguish between the two populations. In epilepsy patient plasmas, we found that concentrations of anti-inflammatory sICAM5 are reduced (*p* = 0.002) and pro-inflammatory IL-1β, IL-2, and IL-8 are elevated. TARC (thymus and activation regulated chemokine, CCL17) concentrations trend high. In contrast, levels of BDNF and a variety of other pro-inflammatory mediators are not altered. Based on *p*-value and ROC analysis, we find that the ratio of TARC/sICAM5 discriminates accurately between patients and controls, with an ROC Area Under the Curve (AUC) of 1.0 (*p* = 0.034). In conclusion, we find that the ratio of TARC to sICAM5 accurately distinguishes between the two populations and provides a statistically and mechanistically compelling candidate blood biomarker for drug resistant epilepsy.

## Introduction

Epilepsy affects up to 1% of the world’s population (Hauser et al., 1993). There are currently no definitive chemical diagnostic biomarkers in blood, so diagnosis is based on a sometimes expensive synthesis of clinical observation, radiology, neuro-psychological testing, and interictal and ictal EEG studies (Engel, 2001, 2011; England et al., 2009). Past attempts to solve this problem have focused on changes in blood concentrations of neuroendocrine hormones, markers of central nervous system tissue (CNS) injury, and more recently inflammation (Abbott et al., 1980; Palmio et al., 2008). In recent rodent studies, pro-inflammatory and cellular immune processes have been invoked as significant mediators of seizure activity (Vezzani and Granata, 2005; Maroso et al., 2010). This focus on inflammation has been productive, with some groups finding altered cytokine levels in epilepsy patient blood samples, including changes that seem to track with successful epilepsy treatment (Lehtimaki et al., 2007, 2010; Alapirtti et al., 2009, 2012; Iyer et al., 2010; Majoie et al., 2010). However, the previously published candidate inflammatory biomarkers are typically produced by non-CNS tissues, so a highly specific blood biomarker for epilepsy, of unambiguous CNS origin, remains to be discovered (Aronica and Crino, 2011).

Here we have focused our attention on the anti-inflammatory soluble fraction of telencephalin (sICAM5), a protein which is produced normally only in the hippocampus and forebrain (Yoshihara and Mori, 1994; Tian et al., 2008). *In vitro*, sICAM5 inactivates T-cells (Tian et al., 2008). T-cells are important in the development of epilepsy in the pilocarpine induced status epilepticus model (Fabene et al., 2008). In the past, relatively insensitive assays for sICAM5 had detected it in a small subset of epilepsy patients (Rieckmann et al., 1998; Jansen et al., 2008). In contrast, we have discovered that plasma sICAM5 is readily detected, and we have therefore reassessed plasma sICAM5 as a biomarker for epilepsy diagnosis using a new, highly sensitive assay.

Consistent with the pro-inflammatory model of drug resistant epilepsy, our data show that patient plasma has low concentrations of the anti-inflammatory sICAM5 and high concentrations of some pro-inflammatory mediators. In addition, we find that the ratio of TARC (thymus and activation regulated chemokine, CCL17) to sICAM5 constitutes a compelling candidate proteomic signature for treatment-resistant epilepsy.

## Materials and Methods

### Patients

We collected plasma samples from in-patients already undergoing epilepsy monitoring for clinical care at the Hospital of the University of Pennsylvania between January and August of 2010. All patients gave informed consent for the study, which was approved in advance by the University of Pennsylvania’s institutional review board (IRB).

The diagnosis of focal epilepsy was confirmed by ictal EEG recordings. The majority of control samples used in this research were purchased from Innovative Research, and additional samples were collected at the National Institutes of Health (NIH) under a separate IRB approved protocol. The NIMH samples were all from individuals who had been first qualified as healthy controls by board certified psychiatrists, and were diagnosed as psychiatrically and physically normal. The commercial plasma samples were from donors, said to be free of physical or psychiatric disease. None of the controls were known to be on antiepileptic drugs.

### Blood collection and plasma preparation

The blood samples collected from epilepsy patients were interictal and not correlated with the timing of patients’ seizures. The samples were anticoagulated using Na-citrate and briefly transported on ice. The plasma was prepared by centrifuging samples at 5,000 × *g* for 15 min at 4°C, and the supernatant solutions were then aliquoted and stored at −80°C.

Blood samples from the NIH were collected at 9 a.m. from control patients also using Na-citrate as the anticoagulant. Following centrifugation, the supernatant solutions were aliquoted and frozen at −80°C. Plasma samples purchased from the two commercial vendors were also anticoagulated with Na-citrate. Differences among sets of controls were not significant (*p* < 0.05) and the controls were grouped together.

### Assay of telencephalin/sICAM5

Levels of immunoreactive Telencephalin/sICAM5 in plasma were measured by sandwich ELISA using electrochemiluminescence detection. Assays were carried out on high bind SECTOR^®^ Imager 6000 reader plates (Meso Scale Discovery, Gaitherburg, MD, USA) as follows. Wells were coated overnight with protein G affinity purified mouse monoclonal anti-human sICAM5 antibody (capture antibody; R&D Systems, Minneapolis, MN, USA; catalog # MAB 1950), 2 μg/mL diluted in phosphate buffered saline (PBS; 25 μL/well). Wells were emptied and then blocked for 2 h with 10% fetal bovine serum (FBS; Invitrogen, Carlsbad, CA, USA) in PBS (PBS-10% FBS). Wells were washed 3× with PBS containing 0.05% tween-20 (PBS-T) and samples were introduced into the wells in a total volume of 100 μL consisting of 25 μL human plasma and 75 μL PBS-5% FBS. ICAM5 standard curves were prepared similarly in buffer containing 25 μL equine plasma (human ICAM5-free; Invitrogen, Carlsbad, CA, USA), to control for the affects of sample matrix. Plates were incubated for 3 h, washed and then incubated for 1 h with biotinylated goat anti-human ICAM5 antibody purified by human ICAM5 affinity chromatography (R&D Systems; catalog #BAF1950; 1 μg/mL in PBS-1% FBS; 25 μL/well). Plates were washed and reacted for 1 h with MSD^®^ SULFO-TAG labeled streptavidin detection reagent (Meso Scale Discovery; catalog# R32AD; 1 μg/mL in PBS containing 1% bovine serum albumin (BSA); 25 μL/well). Plates were washed, treated with the addition of MSD Read Buffer (Meso Scale Discovery; catalog# R92TC; 150 μL/well) and electrochemiluminescence read using a SECTOR^®^ Imager 6000 instrument (Meso Scale Discovery). All incubations were carried out at room temperature with the exception of that for the capture antibody which was carried out at 4°C. The assay was sensitive to less than 0.34 ng/mL as defined by the electrochemiluminescence signal value that was 10× the standard deviation above the mean electrochemiluminescence signal recorded for the 0 ng ICAM5 standard (*N* = 10). The *Z* score for this assay is 0.92 (see Statistics in Materials and Methods for calculation).

### Assay of cytokines and chemokines

Two multiplexed assays for cytokines and chemokines were used for analysis of patient and control plasma samples on the SECTOR^®^ Imager 6000 instrument (Meso Scale Discovery, Gaitherburg, MD, USA). The first of these assays was the Human Pro-Inflammatory 9 Plex Assay (MesoScale catalog #K15007C-4) for the measurement of IL-2, IL-8, IL-12p70, IL-1β, GM-CSF, IFN-γ, IL-6, IL-10, and TNF-α. The second of these assays was the Human Chemokine 9 Plex Assay (MesoScale catalog #K15001C-1) for the measurement of Eotaxin, Eotaxin-3,MIP-1β, MCP-1, MCP-4, TARC, IP-10, IL-8, and MDC, and was used. The samples were added to plates that were pre-coated with capture antibodies for the specific cytokines. The plates was sealed and shaken at room temperature for 2 h. The plates was washed in PBS + 0.05% Tween-20 and detection antibody solution (1× or 1 μg/mL) was added. The plates were once again sealed and set to shake at room temperature for 2 h. The plate was then washed once more in PBS + 0.05% Tween-20. Read buffer was added at a 2× concentration and the plate was read on the SECTOR^@^ 6000 Imager.

Levels of immunoreactive BDNF in plasma were measured in a manner similar to sICAM5 using antibodies and BDNF standard protein provided in the R&D Systems human BDNF ELISA Development Kit (catalog # DY248). Detection was by electrochemiluminescence using the MSD^®^ SULFO-TAG labeled streptavidin detection reagent and the SECTOR^®^ Imager 6000. The assay was sensitive to less than 0.08 ng/mL, as defined by the electrochemiluminescence signal value, which is 10× the standard deviation above the mean electrochemiluminescence signal recorded for the 0 ng BDNF standard (*N* = 10).

### Statistical analysis

Differences in levels between epilepsy samples and normal controls were calculated using a two-tailed *t*-test, except where indicated, and taken to be significant at the *p* ≤ 0.05 level, or ≥2 standard deviations (SD) from the mean (SD ≥ 2.0), as appropriate. Receiver Operating Condition (ROC) curves were calculated and plotted (Srivastava et al., 2011). *Z*-scores for the new sICAM5 and BDNF assays were calculated from the equation *Z* = 1−3[(SD,1 + SD,2)/(μ2−μ1)], where SD,1 is one standard deviation from the mean of the blank; SD,2 is one standard deviation from the mean of the signal; μ1 is the mean of the blank and μ2 is the mean of the signal. Quality control was based on the following criteria: (i) visual inspection of the multiplexed signals revealed no defects; (ii) signal to noise ratio was >3; (iii) duplicate assays agreed to within a coefficient of variation (CV) of <2.5%; (iv) all data were greater than the lower limit of detection (LLOD).

## Results

### Characteristics of the subjects

All of the epilepsy patients were cognitively capable of giving informed consent and all had partial onset seizures. The time from seizure to blood draw was reliably known in six patients (average 23.3 h, range: 3–53). The patients’ average age was 43.4 years (range 26–62), 50% were female, their average duration of epilepsy was 11 years (range 1–25), and their average number of seizures in the day prior to blood draw was 21.1 (range 0–15).

Ninety percent of the epilepsy subjects had a left hemispheric onset. The epilepsy patients had chronically been administered a variety of antiepileptic drugs. One epilepsy patient had a diagnosis of lupus and had received both Cellcept and Plaquenil and another received montelukast sodium for asthma. No other patients were known to be taking any potentially immune modulating therapy (Table 1).

**Table 1:** **Epidemiology and clinical characteristics of the epilepsy patients**.

Patient	Age	Gender	Duration of epilepsy (years)	AEDs	Location
1	40	M	18	ZNS, PHT	Left fronto-temporal
2	27	M	8	LVT, ZNS	Left fronto-temporal
3	54	F	19	TPM, CZP	Left fronto-temporal
4	62	M	1	OXC, CZP, PGB	Left fronto-temporal
5	37	F	Unknown	LVT, TPM, LCM	Left fronto-temporal
6	37	F	25	PHT, LZP	Right frontal, poorly localized
7	48	M	Unknown	PHT, OXC, LVT, TPM	Left temporal
8	52	M	3	LVT, PHB, TPM	Left hemisphere
9	51	F	11	OXC, ZNS	Left temporal
10	26	F	3	LTG, ZNS	Left temporal

Controls were obtained from the NIH (*n* = 10) and from Innovative Research (*n* = 20). Although the epidemiology and epilepsy status of the controls are unknown, none were collected from an epilepsy enriched population.

### Measurement of sICAM5 in plasma from epilepsy patients

**Figure 1:**
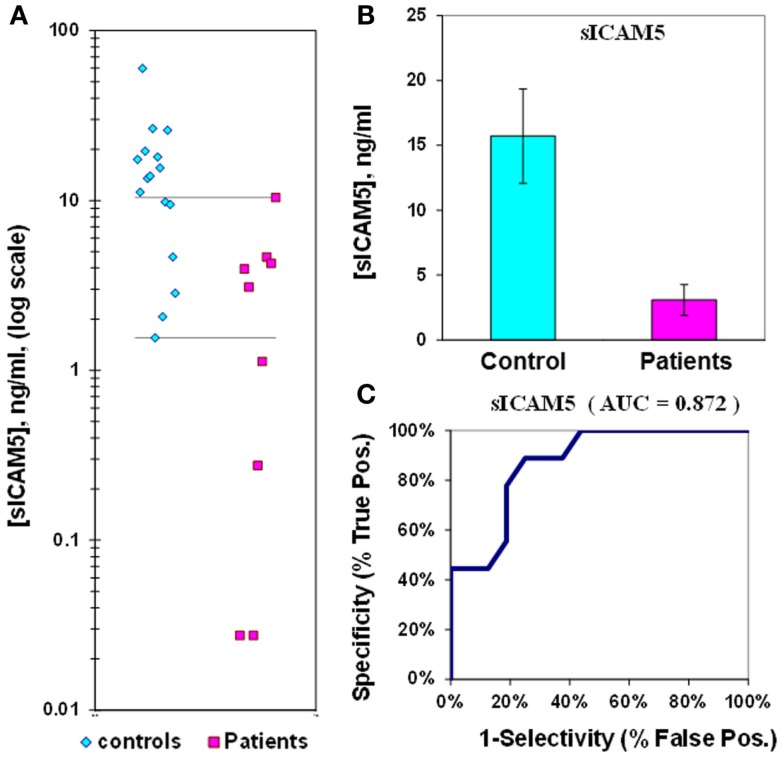
**Assay of sICAM5 in plasma from epilepsy patients and controls**. **(A)** Dot-plot of sICAM5 concentrations in plasma (square, patients; diamond, controls). For values between the horizontal lines, the assay does not accurately discriminate between patients and controls. **(B)** Bar graph and error calculation for data in Part **(A)**. Difference is significant for *p* = 0.002. **(C)** ROC curve for data in Part a, showing an area under the curve (AUC) value of 0.87.

The median value of sICAM5 in control plasmas is 16 ± 4 (ng/mL) while in epilepsy plasmas the value is 3.1 ± 1.2 (ng/mL). This represents a 5.1-fold reduction in sICAM5 in epilepsy plasmas compared to control plasmas. This difference between patients and controls is also highly significant (*p* = 0.002). There is some overlap of values for plasma sICAM5 between epilepsy patients and controls, and the ROC Area Under the Curve (AUC) of 0.87 (Figure 1; Table 2).

**Table 2:** **Levels of cytokines and chemokines in plasma that significantly distinguish between epilepsy patients and controls**.

Analyte	Units	Analyte concentration		Ratio (E/C)	*p*-Value	AUC	*n* Epi	*n* con
		Epilepsy	Controls	
sICAM5	ng/mL	3.1 ± 1.2	16 ± 4	↓ 5.1	**0.002**	**0.87**	9	16
IL-1β	pg/mL	0.2 ± 0.1	0.1 ± 0.0	↑ 3.4	**0.018**	0.73	10	26
IL-6	pg/mL	3.1 ± 1.3	1.1 ± 0.2	↑ 2.8	0.067	**0.81**	10	26
IL-2	pg/mL	0.7 ± 0.2	0.3 ± 0.0	↑ 2.6	**0.013**	0.79	10	26
IFN-γ	pg/mL	1.4 ± 0.3	0.7 ± 0.1	↑ 2.1	**0.014**	0.76	10	26
TARC	pg/mL	148 ± 44	77 ± 13	↑ 1.9	0.068	0.72	8	9
IL-12p70	pg/mL	0.9 ± 0.2	1.6 ± 0.3	↓ 1.7	**0.038**	0.59	10	24
IL-8*	pg/mL	3.8 ± 0.5	2.7 ± 0.2	↑ 1.4	**0.020**	0.75	10	27
TARC/sICAM5		(94 ± 41)·10^3^	(7.2 ± 2.5)·10^3^	↑ 13.0	**0.034**	**1.00**	6	8
IL-6/sICAM5		(2.9 ± 1.9)·10^3^	(0.2 ± 0.1)·10^3^	↑ 14.9	0.087	**0.90**	7	16
IL-8/sICAM5*		(4.0 ± 3.0)·10^3^	(0.5 ± 0.2)·10^3^	↑ 8.2	0.125	**0.88**	7	17

*IL-8 is the average of the values from the chemokine and the cytokine plates for each patient.

*Bolded *p*-values are 0.05. Bolded AUC indicate >0.8*.

### Measurement and composite ratios of cytokines and chemokines in plasma from epilepsy patients

We used the MesoScale platform to measure 17 additional analytes. Five of these analytes had *p*-values < 0.05 when comparing levels in epilepsy and control plasmas. In the order of decreasing fold elevation, these included the following: IL-1β (3.5×) > IL-2 (2.9×) > IFN-γ (2.2×) > IL-8(1.4×) > IL-12p70 (1.7× lower; Table 2). Data were also obtained for TNF-α, MDC, BDNF, IL-10, GM-CSF, MCP-1, MIP-1β, MCP-4, IP-10, Eotaxin-3, and Eotaxin, but were not significant (Table 3).

**Table 3:** **Levels of cytokines and chemokines in plasma that do not significantly distinguish between epilepsy patients and controls**.

Analyte	Units	Analyte concentration		Ratio (E/C)	*p*-Value	AUC	*n* Epi	*n* con
		Epilepsy	Controls	
TNF-α	pg/mL	7.7 ± 2.5	5.5 ± 0.4	↑ 1.4	0.188	0.59	10	25
MDC	ng	2.6 ± 0.4	2.2 ± 0.2	↑ 1.2	0.151	0.77	8	9
BDNF	ng	0.8 ± 0.2	1.0 ± 0.2	↓ 1.2	0.290	0.61	9	10
IL-10	pg/mL	1.4 ± 0.2	1.8 ± 0.3	↓ 1.2	0.161	0.50	10	25
GM-CSF	pg/mL	1.2 ± 0.3	1.4 ± 0.4	↓ 1.2	0.303	0.56	10	24
MCP-1	pg/mL	238 ± 41	219 ± 13	↑ 1.1	0.330	0.65	8	9
MIP-1β	pg/mL	66.2 ± 11.7	58 ± 6	↑ 1.1	0.268	0.65	8	9
MCP-4	pg/mL	388 ± 81	367 ± 67	↑ 1.1	0.419	0.52	8	9
IP-10	pg/mL	189 ± 47	207 ± 38	↓ 1.1	0.377	0.56	8	9
Eotaxin-3	pg/mL	5.9 ± 0.8	6.4 ± 1.1	↓ 1.1	0.348	0.51	8	9
Eotaxin	pg/mL	456 ± 128	525 ± 105	↓ 1·1	0.334	0.55	8	9

Similar to result of sICAM5, none of these tested analytes discriminated perfectly between the two populations. Therefore, we attempted to create a composite biomarker with sICAM5 as one of the elements. IL-6, IL-8, and TARC were included in this analysis because of their high baseline concentrations, statistical trend toward higher values in epilepsy patient plasma, and in the case of IL-6, solid literature showing altered plasma concentration in the epilepsy population.

**Figure 2:**
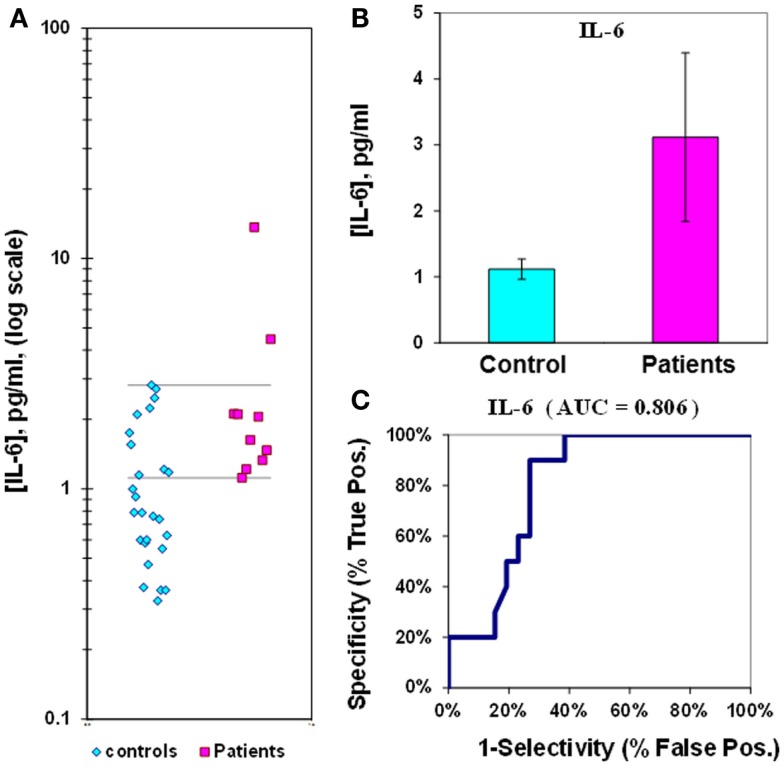
**Assay of IL-6 in plasma from epilepsy patients and controls**. **(A)** Dot-plot of IL-6 concentrations in plasma (square, patients; diamond, controls). For values between the horizontal lines, the assay does not accurately discriminate between patients and controls. **(B)** Bar graph and error calculation for data in Part **(A)**. Difference is not significant for *p* = 0.067. **(C)** ROC curve for data in Part a, showing an area under the curve (AUC) value of 0.81.

**Figure 3:**
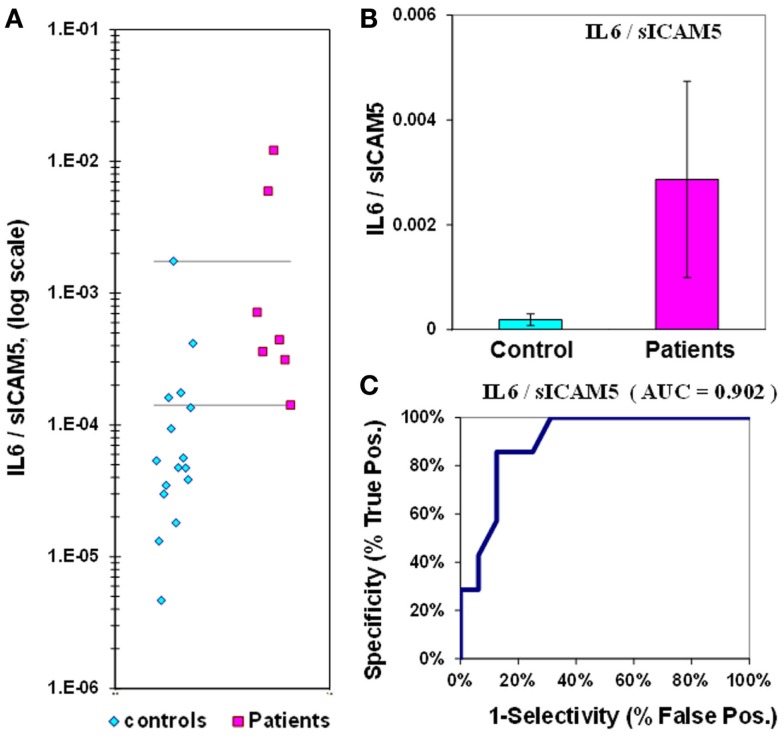
**IL-6/sICAM5 ratio in plasma from epilepsy patients and controls**. **(A)** Dot-plot of IL-6/sICAM5 concentrations in plasma (square, patients; diamond, controls). For values between the horizontal lines, the assay does not accurately discriminate between patients and controls. **(B)** Bar graph and error calculation for data in Part **(A)**. Difference is not significant, with *p* = 0.087. **(C)** ROC curve for data in Part **(A)**, showing an area under the curve (AUC) value of 0.90.

The median value for IL-6 in control plasmas is 1.1 ± 0.2 pg/mL, while in epilepsy plasmas the value is 3.1 ± 1.3  pg/mL. The IL-6 *p*-value is 0.067, and the ROC curve has an AUC of 0.81 (Figure 2; Table 2). When IL-6 is analyzed as a composite ratio to sICAM5, the IL-6/sICAM5 ratio can discriminate between epilepsy and control plasmas by a factor of 14.9-fold. However, the *p*-value is only 0.087, and the ROC curve for the IL-6/ICAM5 ratio has an AUC value of 0.90 (Figure 3; Table 2).

**Figure 4:**
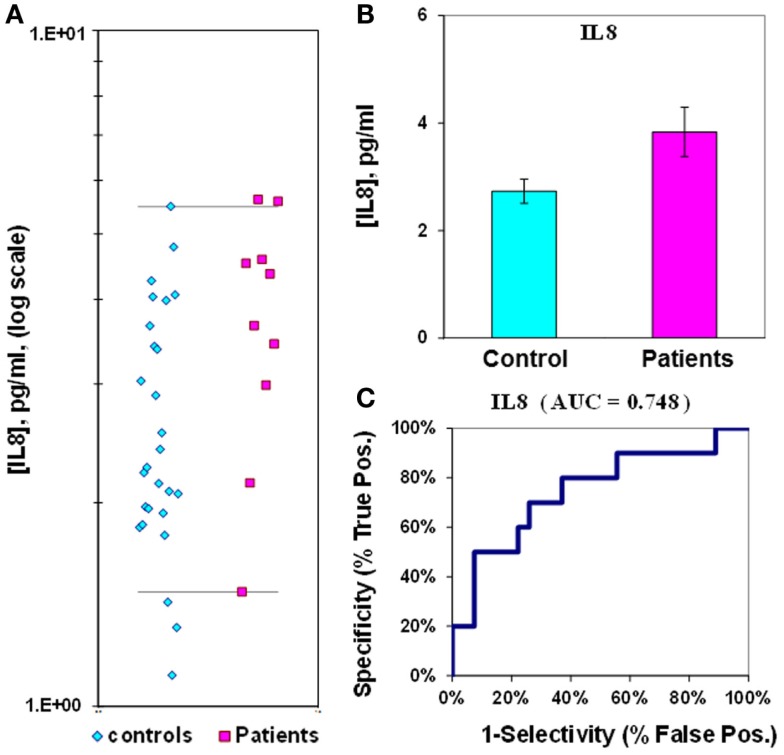
**Assay of IL-8 in plasma from epilepsy patients and controls**. **(A)** Dot-plot of IL-8 concentrations in plasma (square, patients; diamond, controls). For values between the horizontal lines, the assay does not accurately discriminate between patients and controls. **(B)** Bar graph and error calculation for data in Part **(A)**. Difference is 1.4-fold, and significant for *p* = 0.020. **(C)** ROC curve for data in Part **(A)**, showing an area under the curve (AUC) value of 0.75.

**Figure 5:**
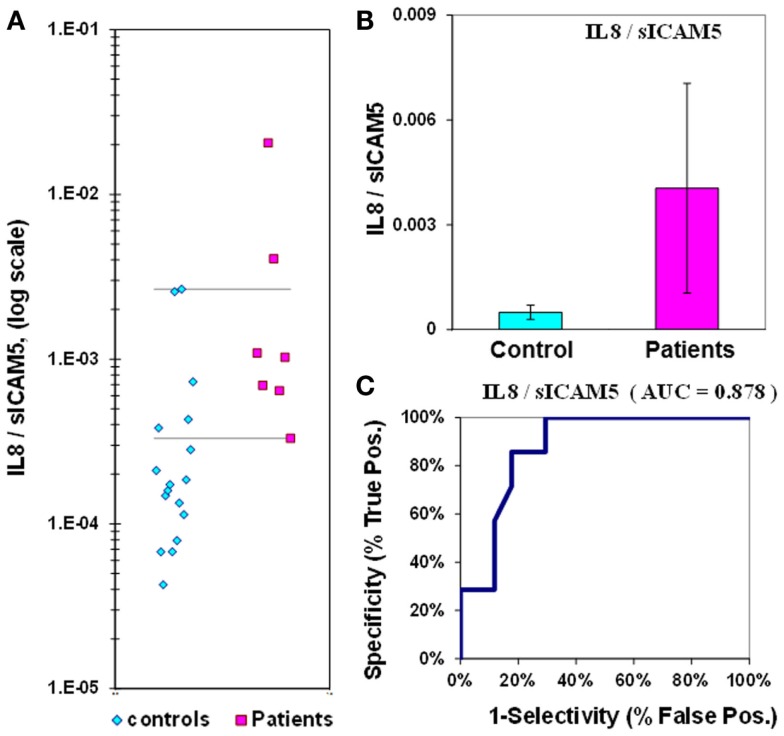
**IL-8/sICAM5 ratio in plasma from epilepsy patients and controls**. **(A)** Dot-plot of IL-8/sICAM5 concentrations in plasma (square, patients; diamond, controls). For values between the horizontal lines, the assay does not accurately discriminate between patients and controls. **(B)** Bar graph and error calculation for data in Part **(A)**. Difference not significant, with *p* = 0.125. **(C)** ROC curve for data in Part **(A)**, showing an area under the curve (AUC) value of 0.88.

The median value for IL-8 in control plasmas is 2.7 ± 0.2 pg/mL, while in epilepsy plasmas the value is 3.8 ± 0.5 pg/mL; the difference between medians for IL-8 is only 1.4-fold. The *p*-value is 0.02 and the ROC curve has an AUC value of 0.75 (Figure 4; Table 2). If IL-8 is analyzed as a composite ratio to sICAM5, the IL-8/sICAM5 ratio can discriminate between epilepsy and control plasmas by a factor of 8.2-fold (Figure 5). However, the difference is not significant, based on the *p*-value of 0.125. Consistently, the substantial overlap evident in the dot-plot results in an ROC curve for the IL-8/sICAM5 ratio that has an AUC value of only 0.88 (Figure 5; Table 2).

**Figure 6:**
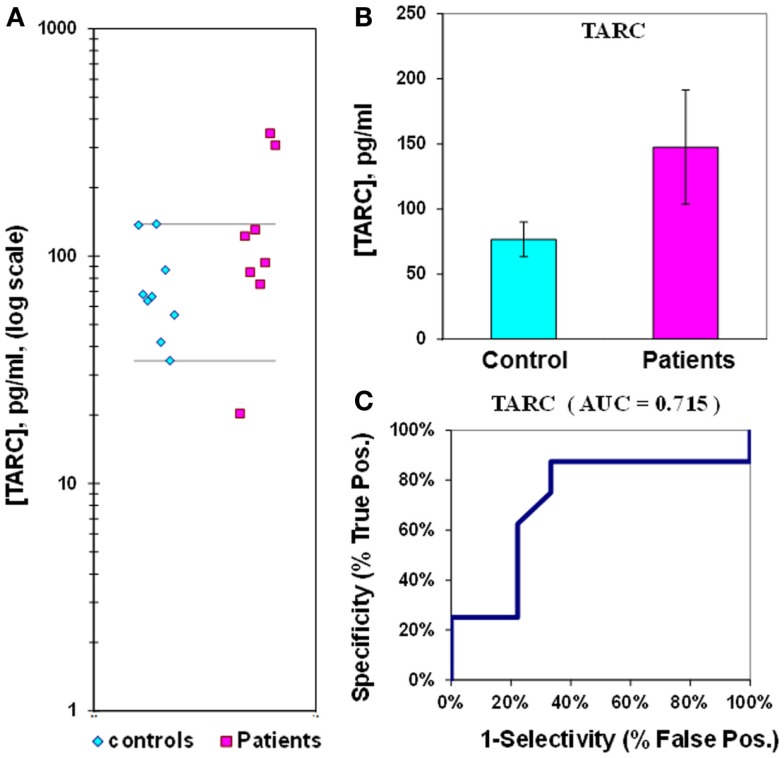
**Assay of TARC in plasma from epilepsy patients and controls**. **(A)** Dot-plot of TARC concentrations in plasma (square, patients; diamond, controls). For values between the horizontal lines, the assay does not accurately discriminate between patients and controls. **(B)** Bar graph and error calculation for data in Part **(A)**. Difference is not significant for *p* = 0.068. **(C)** ROC curve for data in Part **(A)**, showing an area under the curve (AUC) value of 0.72.

**Figure 7:**
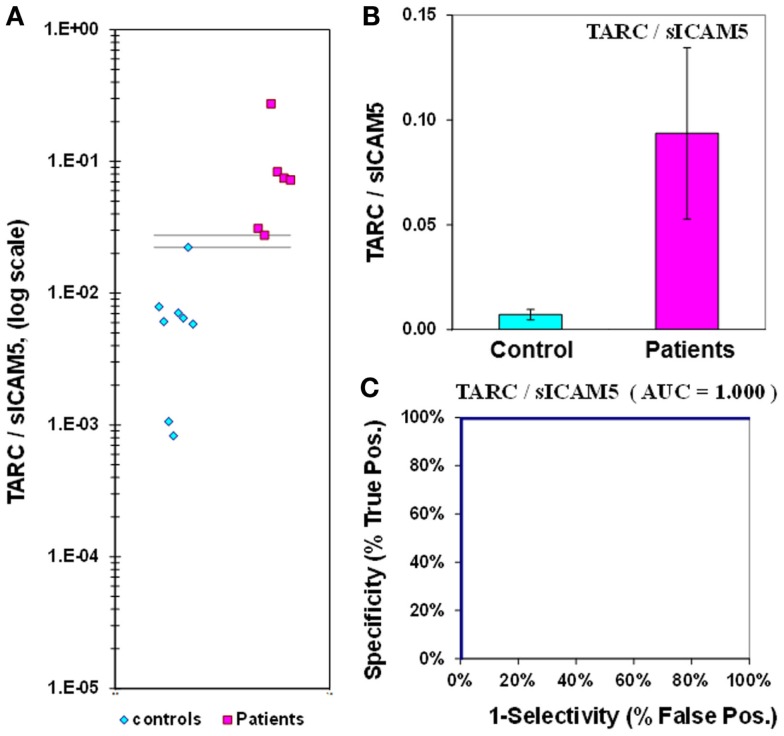
**TARC/sICAM5 ratio in plasma from epilepsy patients and controls**. **(A)** Dot-plot of TARC/sICAM5 concentrations in plasma (square, patients; diamond, controls). There are no values between the horizontal lines, indicating the assay accurately discriminates between patients and controls. **(B)** Bar graph and error calculation for data in Part **(A)**. Difference is 13-fold, and is significant for *p* = 0.034. **(C)** ROC curve for data in Part **(A)**, showing an area under the curve (AUC) value of 1.00.

The mean level of the chemokine TARC in control plasma is 77 ± 13 pg/mL, compared to 148 ± 44 pg/mL in epilepsy patient plasma (Figure 6; Table 2). Epilepsy patient concentrations are 1.9-fold elevated in plasma TARC compared to control patients, though this difference is not significant (*p* = 0.068). The ROC analysis indicates that the AUC is 0.72 (Figure 6; Table 2). However, we found that the TARC/sICAM5 ratio is 13-fold higher in epilepsy patients than controls (Figure 3; Table 2). The difference is significant (*p* = 0.034), and the ROC curve shows that the AUC is 1.00 (Figure 7; Table 2), suggesting a robust statistical relationship that distinguished epilepsy patients from controls.

## Discussion

In this paper we show that in drug resistant epilepsy patients, the plasma concentration of sICAM5, a forebrain-derived inhibitory T-cell regulator, is low while the concentrations of some pro-inflammatory cytokines and chemokines are high. These data lend support to the hypothesis that drug resistant epilepsy is associated with inflammatory changes in plasma.

The ratio of the concentrations of two T-cell regulatory proteins, TARC and sICAM5, is able to discriminate accurately drug resistant epilepsy patients from controls. The TARC/sICAM5 ratio has a high fold-difference between epilepsy and controls (13-fold), strong statistical significance (*p* = 0.034), and a perfect ROC curve (AUC = 1.00; Figure 2; Table 2). We suggest that the plasma TARC/sICAM5 ratio can be considered as a CNS-specific candidate biomarker for drug resistant epilepsy.

### Limitations of the data

We acknowledge some limitations in our dataset. For example, the patient cohort, which is limited in size, consists entirely of focal epilepsy patients, most of whom have left hemispheric foci. In addition, age, gender, and seizure incidence were not taken into account when selecting these patients for analysis. Furthermore, we have no CSF or brain tissue samples to confirm the presence of inflammation in these patients, nor its absence in the controls. Polytherapy with antiepileptic drugs was ubiquitous for the studied patients, and it is possible that some or all of the drugs could have affected the studied parameters. (Beghi and Shorvon, 2011) However, because this study lacks any epilepsy patients on monotherapy or no therapy, delineating each drug’s effect on the outcomes measured was beyond the capacity of this study. Finally, two patients had received immunomodulatory drugs, which could have confounded the findings. The same concerns could also extend to the controls, which were obtained from two independent sources. However, we suggest that the very generality of both the patient and control cohorts might be a strength rather than a limitation of this study. Despite the limitations of both cohorts, the composite TARC/sICAM5 ratio has high fidelity to the epilepsy diagnosis.

### CNS source of sICAM5

ICAM5 is also known as telencephalin because of its exclusive location in the telencephalon. It is specifically excluded from all γ-aminobutyric acid (GABA)-ergic interneurons at all stages of development and is only present on glutamatergic neurons (Yoshihara et al., 1994; Benson et al., 1998). ICAM5 is first expressed around birth, both in mouse and in man, when dendritic outgrowth and branching, spine formation and synapse formation are initiated in the forebrain, and it plays an active role in synapse formation (Yoshihara et al., 1994; Arii et al., 1999; Furutani et al., 2007). ICAM5 also interacts with LFA-1/α_1_β_2_ Integrin, like other members of the ICAM family such as ICAM1 (Mizuno et al., 1997). Brain microglia have LFA-1/α_1_β_2_ Integrin on their cell surfaces and are activated by exposure to ICAM5 (Mizuno et al., 1999). Some have suggested that ICAM5 serves a critical role in effective CNS inflammation (Kipnis et al., 2004; Ziv et al., 2006; Schwartz and Kipnis, 2011).

The only known source for soluble ICAM5 is from the cleavage of membrane bound ICAM5 by matrix metalloproteinases, and there are no described splice variants of ICAM5 (Conant et al., 2010). The production of sICAM5 is known to occur during dendritic growth or glutamate activation (Tian et al., 2000, 2007). The mechanism by which sICAM5 escapes the extracellular fluid and crosses the blood brain barrier to enter the plasma has yet to be elucidated. Since published CSF concentrations of sICAM5 are higher than the plasma concentrations we found, it appears that there is a homeostatic concentration gradient favoring transfer from the CSF to the plasma (Lindsberg et al., 2002). Diffusion might be the simplest explanation for the presence of sICAM5 in plasma.

The low sICAM5 concentration in epilepsy patient plasma could help perpetuate the CNS pro-inflammatory environment through a dearth of negative feedback (Lindsberg et al., 2002). There is precedent for this type of ICAM inflammation feedback loop in another tissue type, alveolar epithelial lung cells. In these cells, the parent ICAM1 is critical to translocation of leukocytes for maintaining immunity, while the cleavage product sICAM1 is part of a negative feedback loop that moderates leukocyte translocation through competition for β_2_ integrin binding (Kusterer et al., 1998; Mendez et al., 2008). If the regulation of sICAM5 follows this type of ICAM1 pattern, feedback could occur through inhibition of T-cell activation and translocation, a mechanism consistent with our findings of disease specific pro-inflammatory alterations in the T-cell regulatory proteins TARC and sICAM5. Further investigation of this relationship might yield mechanistic insights into the pathobiology underlying not only the physical manifestations of epilepsy, but also the functional consequences of the ongoing disease.

### TARC and other non-CNS source inflammatory mediators as biomarkers of epilepsy

Thymus and activation regulated chemokine (TARC/CCL17) is produced by multiple immune cell types and is thought to be a T-cell chemokine (Imai et al., 1996, 1997). Our data showed only a trend toward TARC elevation in epilepsy patients (*p* = 0.06); however, in combination with sICAM5, the result was significant. It is therefore possible that the two analytes may be reflective of the same T-cell pro-inflammatory process.

Previously, cytokines and chemokines had been found to be elevated in epilepsy patients, including high levels of IL-6 postictally (Bauer et al., 2009). The mean values presented in this paper differ from some previously published data examining epilepsy patients’ blood cytokine levels (Peltola et al., 2000). This is likely due to two different factors. First, the Peltola group used different collection methods assaying blood soon after the seizure and surveying a more heterogeneous population. Second, this study uses more precise assays as evidenced by smaller error values. For these reasons, it is difficult to compare the two studies directly. The higher plasma levels observed in this study of IL-6, IL-1β, IL-2, and IL-8, also support the concept that innate immunity is chronically activated in epilepsy.

Interestingly there are also several reports of direct effects of cytokines on excitability of brain tissue (Balosso et al., 2008; Vezzani et al., 2008; Galic et al., 2012). For example, one study delineates the relative attenuation of inhibitory neurotransmission distal to inflamed supporting cells (Ortinski et al., 2010). This imbalance between excitation and inhibition could cause seizures.

If inflammation causes seizures then inflammatory mediators are in the pathway of seizure generation. Plasma sampling of CNS origin sICAM5 might then be used as a biomarker for predicting important aspects of epilepsy care, such as recurrence, efficacy of a new medicine, or perhaps even when a seizure might occur (Vezzani and Friedman, 2011). Future studies should explore this possibility using postictal plasma sampling, expecting up regulation of inflammation peri-ictally. In addition, a rigorous, larger scale prospective study should be initiated to validate the discovery of the TARC/sICAM5 ratio as a diagnostic tool for intractable epilepsy.

In conclusion, this study has identified the CNS origin sICAM5 as an anti-inflammatory plasma protein that is low in epilepsy patient plasma. In addition, the ratio of pro-inflammatory TARC to anti-inflammatory sICAM5 is capable of discriminating epilepsy patients from normal controls. We suggest that this ratio constitutes a CNS-specific candidate biomarker for drug resistant epilepsy. This should be evaluated further with future studies using larger patient cohorts with more diverse seizure localizations, peri- and postictal plasma sampling, and demographic and antiepileptic drug matched controls.

## Conflict of Interest Statement

Three authors would like to disclose potential conflicts of interest. Peter B. Crino and John R. Pollard are co-inventors on a patent based on the data reported in this paper, and the University of Pennsylvania owns the patent. Additionally, Peter B. Crino, John R. Pollard, Elizabeth J. Brand, and the University of Pennsylvania formed a company around this patent primarily for the purpose of applying for small business grants in order to advance the research. The company currently has no monetary value. All other co-authors, Ofer Eidelman, Gregory P. Mueller, Christopher T. Anderson, Evren Burakgazi, Sai K. Ivaturi, and Harvey B. Pollard, have no financial conflicts of interest.
